# Cognitive outcomes in multiple sclerosis are shaped by divergent functional connectivity trajectories

**DOI:** 10.1093/braincomms/fcaf489

**Published:** 2025-12-12

**Authors:** Eloy Martinez-Heras, Elisabet Lopez-Soley, Chiara Cabras, Francesc Vivó, Alberto Calvi, Ferran Prados, Salut Alba-Arbalat, José M Cabrera-Maqueda, Maria Teresa Alba, Maria Sepulveda, Albert Saiz, Yolanda Blanco, Elisabeth Solana, Sara Llufriu

**Affiliations:** Neuroimmunology and Multiple Sclerosis Unit, Laboratory of Advanced Imaging in Neuroimmunological Diseases; Hospital Clinic Barcelona, Institut d’Investigacions Biomediques August Pi i Sunyer (IDIBAPS) and Universitat de Barcelona, Barcelona 08036, Spain; Neuroimmunology and Multiple Sclerosis Unit, Laboratory of Advanced Imaging in Neuroimmunological Diseases; Hospital Clinic Barcelona, Institut d’Investigacions Biomediques August Pi i Sunyer (IDIBAPS) and Universitat de Barcelona, Barcelona 08036, Spain; Neuroimmunology and Multiple Sclerosis Unit, Laboratory of Advanced Imaging in Neuroimmunological Diseases; Hospital Clinic Barcelona, Institut d’Investigacions Biomediques August Pi i Sunyer (IDIBAPS) and Universitat de Barcelona, Barcelona 08036, Spain; Neuroimmunology and Multiple Sclerosis Unit, Laboratory of Advanced Imaging in Neuroimmunological Diseases; Hospital Clinic Barcelona, Institut d’Investigacions Biomediques August Pi i Sunyer (IDIBAPS) and Universitat de Barcelona, Barcelona 08036, Spain; Neuroimmunology and Multiple Sclerosis Unit, Laboratory of Advanced Imaging in Neuroimmunological Diseases; Hospital Clinic Barcelona, Institut d’Investigacions Biomediques August Pi i Sunyer (IDIBAPS) and Universitat de Barcelona, Barcelona 08036, Spain; UCL Hawkes Institute. Department of Neuroinflammation, UCL Queen Square Institute of Neurology, Faculty of Brain Science, University College of London, London WC1N 3BG, UK; Centre for Medical Image Computing (CMIC), Department of Medical Physics and Bioengineering, University College London, London WC1E 6BT, UK; E-Health Center, Universitat Oberta de Catalunya, Barcelona 08018, Spain; Neuroimmunology and Multiple Sclerosis Unit, Laboratory of Advanced Imaging in Neuroimmunological Diseases; Hospital Clinic Barcelona, Institut d’Investigacions Biomediques August Pi i Sunyer (IDIBAPS) and Universitat de Barcelona, Barcelona 08036, Spain; Neuroimmunology and Multiple Sclerosis Unit, Laboratory of Advanced Imaging in Neuroimmunological Diseases; Hospital Clinic Barcelona, Institut d’Investigacions Biomediques August Pi i Sunyer (IDIBAPS) and Universitat de Barcelona, Barcelona 08036, Spain; Neuroimmunology and Multiple Sclerosis Unit, Laboratory of Advanced Imaging in Neuroimmunological Diseases; Hospital Clinic Barcelona, Institut d’Investigacions Biomediques August Pi i Sunyer (IDIBAPS) and Universitat de Barcelona, Barcelona 08036, Spain; Neuroimmunology and Multiple Sclerosis Unit, Laboratory of Advanced Imaging in Neuroimmunological Diseases; Hospital Clinic Barcelona, Institut d’Investigacions Biomediques August Pi i Sunyer (IDIBAPS) and Universitat de Barcelona, Barcelona 08036, Spain; Neuroimmunology and Multiple Sclerosis Unit, Laboratory of Advanced Imaging in Neuroimmunological Diseases; Hospital Clinic Barcelona, Institut d’Investigacions Biomediques August Pi i Sunyer (IDIBAPS) and Universitat de Barcelona, Barcelona 08036, Spain; Neuroimmunology and Multiple Sclerosis Unit, Laboratory of Advanced Imaging in Neuroimmunological Diseases; Hospital Clinic Barcelona, Institut d’Investigacions Biomediques August Pi i Sunyer (IDIBAPS) and Universitat de Barcelona, Barcelona 08036, Spain; Neuroimmunology and Multiple Sclerosis Unit, Laboratory of Advanced Imaging in Neuroimmunological Diseases; Hospital Clinic Barcelona, Institut d’Investigacions Biomediques August Pi i Sunyer (IDIBAPS) and Universitat de Barcelona, Barcelona 08036, Spain; Neuroimmunology and Multiple Sclerosis Unit, Laboratory of Advanced Imaging in Neuroimmunological Diseases; Hospital Clinic Barcelona, Institut d’Investigacions Biomediques August Pi i Sunyer (IDIBAPS) and Universitat de Barcelona, Barcelona 08036, Spain

**Keywords:** multiple sclerosis, cognition, functional connectivity, graph theory, brain atrophy

## Abstract

Cognitive impairment in people with multiple sclerosis (pwMS) is highly heterogeneous, highlighting the need to better understand the underlying pathophysiological processes of cognitive decline and the brain’s mechanisms for adapting to MS damage. This longitudinal study explores functional connectivity (FC) changes and their relationship with cognitive trajectories over seven years in pwMS. We aimed to determine whether individuals with cognitive decline exhibited different FC patterns compared to those with stable cognitive performance. For this purpose, we analysed data from 58 pwMS, including cognitive assessments using the Rao’s battery and functional MRI at two-time points with an interval of seven years. Cognitive worsening was defined as 25% decline in global cognitive scores. Graph-based networks metrics, including global and node-based strength, efficiency and clustering coefficient, alongside regional normalized grey matter (GM) volumes, were computed using MRI. We used mixed effect regression models with random subject-specific intercepts to explore FC and GM volume differences and the association between FC and cognition. The cohort was predominantly female (78%), with a mean age of 46.8 years and a median disease duration of 11.6 years. We found a significant group-by-time interaction, patients with cognitive decline showed reductions in node strength (21.1% of nodes), local efficiency (64.4%), and clustering coefficient (85.5%) particularly in the deep GM and parietal cortex at follow-up, and reduced global graph metrics. In contrast, the cognitively stable group exhibited increased node strength (15.8%) and local efficiency (5.3%), mainly in the temporal and prefrontal cortices. Both groups showed reduced GM volume in 84.2 and 79% of regions at follow-up, respectively. Several links were found between FC changes and cognitive performance. Findings confirm distinct FC trajectories in pwMS associated with their ability to cope with structural damage, impacting cognitive outcomes at mid-term. These findings indicate that patients with stable cognitive performance may engage compensatory network reorganization processes, which could mitigate the progression of cognitive decline.

## Teaser

Martinez-Heras, *et al*., described longitudinal functional connectivity in multiple sclerosis using graph metrics. They identified distinct trajectories linked to cognitive performance, supporting two mechanisms of brain adaptation. Patients with cognitive impairment showed reduced network segregation and integration, suggesting a maladaptive rather than compensatory response to brain damage

## Graphical Abstract

**Graphical Abstract:**
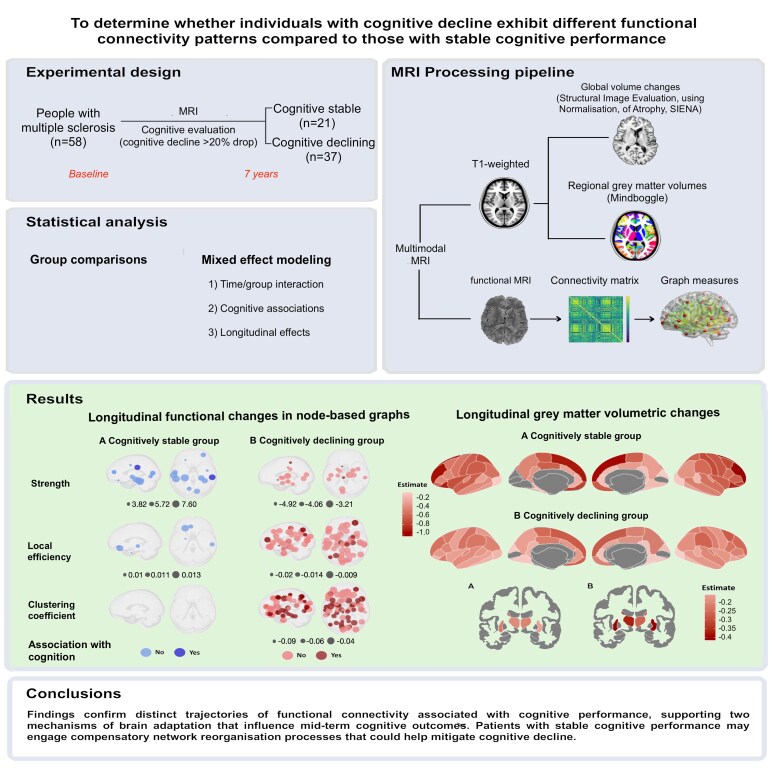

## Introduction

Cognitive impairment is a major manifestation of multiple sclerosis (MS), affecting daily functioning and quality of life.^1^ Its course is highly heterogeneous among patients, while some people with MS (pwMS) maintain cognitive abilities, others develop cognitive deficits that may progress non-linearly.^2^ The patterns of cognitive decline predominantly affect information processing speed, and episodic memory, as well as semantic fluency, and visuospatial memory.^3^ Cognitive impairment in pwMS is multifactorial, resulting from the interplay between demyelination and neurodegeneration processes along with pathophysiological changes affecting critical brain networks supporting cognition.^1^ There is still no effective therapy for cognitive disabilities,^4^ hence it is crucial to understand the pathophysiological mechanisms underlying cognitive decline, and how the brain can adapt to MS damage. Similarly important is the investigation on how these adaptive responses can eventually become depleted following cumulative structural damage, leading to cognitive dysfunction.

It is well recognized that cognitive function relies on a complex network of structurally interconnected brain regions that support a highly dynamic functional network.^1,4^ Functional connectivity (FC), a measure of the statistical correlation of blood-oxygenation-level-dependent signal time course, has been related to cognition.^1,4^ Brain networks can cope with brain damage through functional and structural reorganization, leading to changes in connectivity patterns.^5^ Grey matter (GM) damage, associated with volume loss in specific highly connected regions, such as thalamus and other multimodal areas,^6,7^ contributes to cognitive disability.^2^ However, the longitudinal relationship between these mechanisms, and their impact on cognitive changes along the course of the disease, is not fully elucidated. It has been hypothesized that in the early stages of the disease, when plasticity mechanisms can adapt to the pathological events, increased FC appears to enable cognitive preservation. But, in a given moment, when the structural network is depleted and FC decreases, cognitive deterioration appears.^1,8,9^ In support of this, studies have described distinct patterns of FC changes at different disease stages and according to clinical phenotypes. Some studies reported increased FC in clinically isolated syndrome^10,11^ or in relapsing-remitting MS and reduced FC in progressive MS,^12^ however these findings were not consistent across studies.^4,12^ Other works, which analysed cognitively relevant networks, such as the default mode or the frontoparietal network, found reduced dynamics in these relevant networks in patients with cognitive impairment.^13^ These discrepancies may be explained by methodological aspects, including the methods used in analysing FC, the cross-sectional design and the categorization of patients based on clinical phenotypes, where the brain changes between them may not be clearly different.^14^ Classifying patients along a disease continuum, for instance, using continuous scores, rather than solely into discrete clinical phenotypes, might help explore how plasticity mechanisms can adapt or maladapt to brain damage that occurs throughout the disease progression. To address this, we set out to describe longitudinal FC changes using a graph theoretical approach in relation to cognitive progression over the course of the disease, and to determine whether patients experiencing cognitive decline exhibit distinct patterns compared to those with stable cognition. By analysing these modifications and exploring their relationship to lesion burden and brain atrophy, we aimed to identify specific functional network changes associated with cognitive trajectories to better understand the underlying mechanisms driving cognitive decline or stability in MS.

## Materials and methods

### Participants

For this longitudinal study, we compiled data from a cohort of patients diagnosed according to the 2017 McDonald criteria^15^ recruited between April 2013 and August 2017 at the MS Unit of the Hospital Clinic Barcelona. We selected 58 pwMS with functional MRI scans and neurologic and cognitive examination at two time points more than five years apart. Participants fulfilled inclusion criteria: age between 18 and 65 years, absence of relapses or corticosteroid treatment in the previous month, stability of the disease-modifying treatment and sufficient proficiency in Spanish to fully understand cognitive evaluation. We collected data on gender, age, disease duration and disease type. All participants had been informed about the nature of the study and provided written consent prior to their participation. The Ethics Committee at the Hospital Clinic Barcelona approved the study (HCB/2009/4905, HCB/2015/0236). All study procedures were performed in accordance with the relevant guidelines and regulations.

### Clinical and cognitive assessment

We used the Expanded Disability Status Scale (EDSS)^16^ to assess global disability at baseline and follow-up. Neurological disability worsening was considered after an increase in EDSS at follow-up of 1.5, 1.0 or 0.5 if baseline EDSS was 0, 1.0 or equal or greater than 5.0.^17^

Cognition was assessed using the Brief Repeatable Battery of Neuropsychological tests (BRB-N),^18^ with parallel versions used when available (i.e. Selective Reminding Test, Symbol Digit Modalities Tests and Paced Auditory Serial Addition Test). The battery evaluates multiple cognitive domains, including verbal and visuospatial learning and memory, attention and information processing speed, working memory, verbal fluency and cognitive flexibility. Raw scores were transformed into *Z*-scores by adjusting for sex, age and education based on the Spanish normative data, and a global composite BRB-N *Z*-score was computed.^19^ We defined cognitive worsening as a decline of 25% from the baseline BRB-N global *Z*-score, considering a drop of >20% clinically significant.^20^

### MRI acquisition and processing

MRI images were acquired on 3 Tesla Magnetom Trio and PRISMA scanners (SIEMENS, Erlangen, Germany) using 32- and 64-channel phased-array head coils. Acquisition parameters can be found in the Supplementary material.

We automatically segmented white matter lesion masks in 3D-MPRAGE and 3D-FLAIR using nnU-Net architecture,^21^ followed by a quality check conducted by an expert neurologist. Subsequently, lesion location was computed using LST-AI,^22^ which classified the lesions into periventricular, juxtacortical and subcortical categories. Then, we applied T1-weighted lesion filling to ensure accurate GM segmentation into 62 cortical brain regions using Mindboggle-101 according to the Desikan–Killiany–Tourville atlas,^23,24^ and 14 subcortical structures using the FSL-FIRST tool^25^ as part of the node parcellation scheme. Longitudinal global volume changes were calculated using the percentage of brain volume change from FSL-SIENA.^26^ Both GM and lesion volumes were normalized for head size.

The resting-state FC was derived following established preprocessing steps,^27^ including slice timing and correction, skull stripping and regression of white matter and cerebrospinal fluid signals to isolate GM activity. A low-pass filter (<0.08 Hz) was applied using FSL tools.^28^ From the preprocessed resting-state images, we extracted the average time series for 76 brain regions. A connectivity adjacency matrix was then generated for each subject by calculating the Pearson correlation coefficient between every pair of regional time series. Finally, we used the absolute values of the correlation matrix to retain only the strength of the relationship, addressing potential drawbacks in calculating network metrics related to the distance or shortest path.^29^

To account for potential confounding factors, we explicitly modelled age and gender as biological covariates in our statistical analysis. To explicitly test for batch effects related to the scanner upgrade, our quality control analysis revealed statistically significant changes in data quality, impacting both the overall image signal-to-noise properties and the temporal signal stability (*P* < 0.05).^30^ Because these metrics served as direct evidence of a scanner-level batch effect, the ComBat harmonization technique was applied to remove these systematic, non-biological effects from all quantitative imaging measures, including both the FC matrices and the normalized GM volumes.^27^

### Graph metrics

Graph metrics were computed using the Brain Connectivity Toolbox, analysing both global and node-based FC properties of the network. At the global level, we calculated average strength, global efficiency and clustering coefficient computed from the corresponding node-based metrics. Node-based metrics included strength (sum of neighbouring link weights), local efficiency (how well a node’s neighbours communicate in the absence of this node) and clustering coefficient (how densely the neighbours are interconnected). These metrics capture segregation and integration mechanisms to characterize the network’s topological properties.^31^

### Statistical analysis

Descriptive data were reported as median and interquartile range, or mean and standard deviation for quantitative variables, and absolute numbers and proportions for qualitative data. We checked for normal distribution using the Shapiro–Wilks test. Differences in baseline demographic, clinical and MRI data between groups were analysed using Student *t*-test or Mann-Whitney U test for continuous variables and Chi-square test for categorical ones. Longitudinal demographic, clinical and cognitive changes were evaluated through mixed effect linear models, except for categorical data, which we used multinomial logistic regression.

MRI longitudinal changes were analysed using mixed effect linear regression models with subject-specific random intercepts, testing (i) global and node-based FC graph metrics, and (ii) regional GM and lesional volumes, with disease duration as a fixed effect. Two main models were employed: one examined time-by-group interaction to identify trajectory differences among groups, the other assessed within-group longitudinal changes. Additional models included adjustments for global GM, white matter lesion volume, and education. Associations between FC metrics and cognition were investigated using node-based metrics as fixed effects, with subject and time as random effects for each patients’ group.

In the group comparison analysis, the false positive errors correction was applied using the Benjamini–Hochberg False Discovery Rate (FDR) procedure when multiple comparisons were conducted, and *P* < 0.05 were considered statistically significant. Statistics were conducted using R language (v. 4.1.2).

## Results

### Demographic, clinical and MRI characteristics

We included 58 pwMS with both baseline and follow-up visits. At baseline, the cohort was mostly female (78%), with a mean age of 46.8 years, and 91% had relapsing–remitting MS. No demographic, clinical, cognitive or educational differences were observed between groups. Table 1 summarizes baseline demographic, clinical and structural MRI characteristics of participants.

**Table 1:** Baseline demographic, clinical and structural MRI characteristics of the study population

	Entire cohort (*n* = 58)	Cognitive groups		
		Stable (*n* = 21)	Declining (*n* = 37)	*P*-value
Age	46.8 (38.3, 53)	46.7 (39.6, 50.5)	47.1 (38.2, 53.9)	0.594^a^
Gender, female (*n*, %)	45 (78)	17 (81%)	28 (76%)	0.892^c^
MS phenotype (*n*, %):				0.999^c^
Relapsing-remitting	53 (91%)	19 (90%)	34 (94%)	
Progressive	5 (9%)	2 (10%)	3 (6%)	
Median EDSS (range)	2.0 (1.0–6.5)	2.0 (1.0–6.5)	2.0 (1.0–6.0)	0.485^a^
Disease duration, years	11.6 (5.9, 16.9)	11.9 (9.3, 17.7)	11.5 (5.7, 16.1)	0.594^b^
Number of previous relapses	4 (2, 5)	4 (3, 7)	3 (2, 5)	0.15^b^
Use of DMT (*n*, %)				0.716^c^
None	15 (26)	6 (29)	9 (24)	
Platform therapies	38 (65)	14 (66)	24 (65)	
High efficacy	5 (9)	1 (5)	4 (11)	
Education (*n*, %):				0.509^c^
Low education	24 (41)	7 (33)	17 (46)	
High education	34 (59)	14 (67)	20 (54)	
GM volume (cm^3^)	723.03 (685.1, 749.6)	730.06 (697.5, 756.1)	706.68 (679.4, 740.8)	0.083^a^
Lesion volume (cm^3^)	5.38 (2.9, 13.5)	4.05 (2.7, 13.2)	7.7 (3.6, 13.7)	0.552^b^
BRB-N, *Z*-score	0.031 (−0.43, 0.44)	−0.399 (−0.87, 0.4)	0.0*92* (−0.28, 0.57)	0.329^a^

Continuous variables are given as median and interquartile range, and qualitative data as numbers and proportion. EDSS, expanded disability status scale; DMT, disease- modifying treatment; BRB-N, Brief Repeatable Battery of Neuropsychological test; GM, normalized GM.

^a^Student *t*-test.

^b^Mann–Whitney U test.

^c^Chi-square test.

After an interval time of 7.4 years (range 5.2–8.3), 37 patients (64%) showed cognitive worsening, while the remaining 21 (36%) were considered cognitively stable. The stable group had higher average *Z*-scores on the BRB-N at follow-up. Seven (33%) participants from the stable group and nine (24%) in the worsening group showed a significant increase in EDSS at follow-up. The cognitively worsening group had a higher percentage of participants evolving to a progressive form of the disease (*Pcorr* = 0.013), and lower performance in both verbal and visuospatial learning and memory, as well as in verbal fluency at follow-up (*Pcorr* < 0.03). For further details, see supplementary Table 1.

### Functional connectivity along the study time

At baseline, global graph theory metrics were similar between pwMS with stable and declining cognitive performance (*Pcorr* > 0.05). There was a significant interaction effect between time (baseline versus follow-up visits) and group (stable versus declining), indicating differential trajectories. Indeed, in the cognitively declining group, FC graph metrics—global strength, global efficiency and clustering coefficient—decreased at follow-up (*P* = 0.04, 0.019 and <0.001, respectively), whereas patients in the stable group showed no significant modifications (*P* > 0.05).

**Figure 1:**
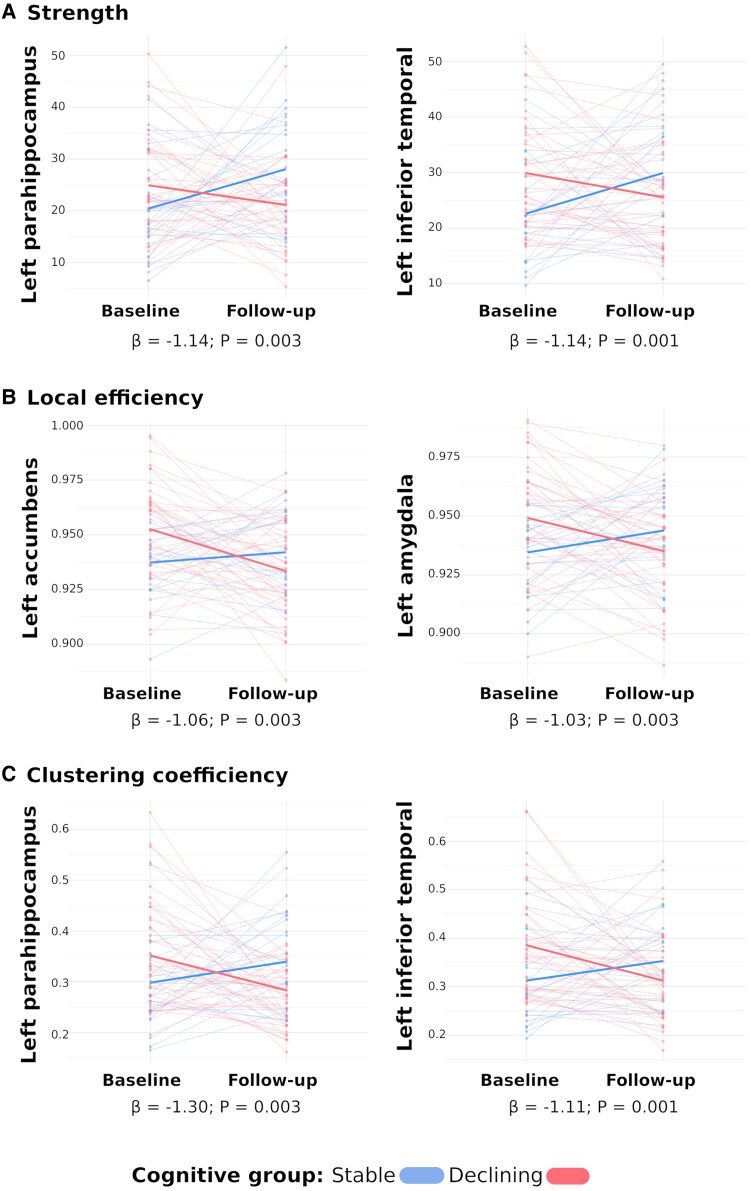
**Nodal functional connectivity values along the time for each cognitive group.** The figure shows longitudinal differences in (**A**) node-based strength, (**B**) local efficiency and (**C**) clustering coefficient between baseline and follow-up for each cognitive group analysed by mixed effect models with subject-specific random intercept. The *y*-axis shows node-based graph metrics and the *x*-axis the time-point (baseline and follow-up). Blue represents cognitively stable patients, while red represents the cognitively declining group. Bold lines indicate probabilistic regression lines. The regions were selected based on the highest statistical significance, as determined by their beta value.

FC nodal properties did not differ between groups at baseline (*Pcorr* > 0.05). When exploring nodal-based metrics trajectories, we identified distinct patterns between patients experiencing cognitive worsening and those who remained stable. Differences were observed in strength in 21 (27.6%) nodes, local efficiency in 65 (85.5%) nodes and clustering coefficient in 30 (39.5%) nodes, with trajectories varying by cognitive group. PwMS in the declining group exhibited reduced connectivity over time, whereas those in the stable group showed stable or increased connectivity. The most significant changes were localized in the left temporal cortex, including parahippocampal (mean ß = −1.1, 95% IC: −1.8, −0.4; *P* = 0.004), middle temporal (mean ß = −1.06, 95% IC: −1.7, −0.4; *P* = 0.003) and inferior temporal cortex (mean ß = −1.06, 95% IC: −1.7, −0.4; *P* = 0.003), as well as the left accumbens (mean ß = −1.05, 95% IC: −1.7, −0.4; *P* = 0.003). (Fig. 1 displays the nodes with the most pronounced changes between groups based on the highest statistical significance, as determined by their beta values).

**Figure 2:**
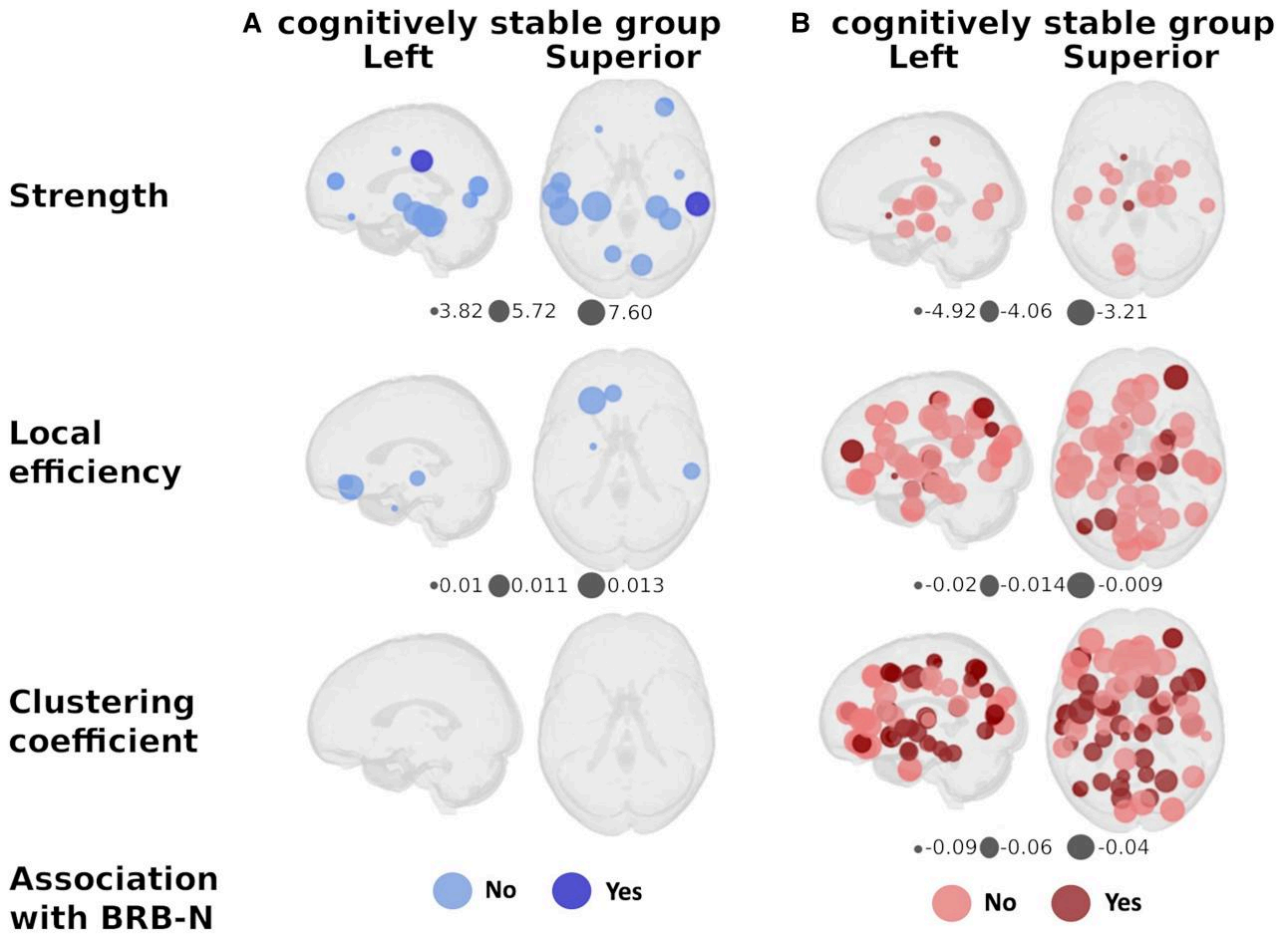
**Functional connectivity changes over study time.** The figures depict longitudinal differences in node-based strength, local efficiency and clustering coefficient for each group, (**A**) cognitively stable and (**B**) cognitively declining patients analysed by mixed effect models with subject-specific random intercept (*P* < 0.05). Circle size reflects the magnitude of beta coefficients; colour indicates direction—blue for positive and red for negative. Dark blue and red highlight nodes where the graph metric is significantly associated with cognition. Figures were generated using *netplotbrain* (netplotbrain.org) running in Python.

When modelling FC modifications over time within each group, the cognitively stable pwMS exhibited increased FC at follow-up, with greater strength in 12 nodes (15.8%) and higher local efficiency in four nodes (5.3%), mainly in the temporal and prefrontal cortex, while other nodes remained unchanged. On the contrary, clustering coefficient did not show any significant changes (*P* < 0.05). Patients showing cognitive worsening displayed reduced strength in 16 nodes (21.1%) and reduced local efficiency in 52 (64.4%) regions, particularly in deep GM and parietal cortex. Additionally, clustering coefficient also decreased in 65 (85.5%) regions, mainly in the temporal, parietal and deep GM (Fig. 2). After adjusting models for normalized GM and lesion volume, results remained unchanged, except for the reduction in the clustering coefficient of the right postcentral cortex, which became not statistically significant (*ß* = −0.132, 95% CI: −0.31, 0.05; *P* = 0.142). Results also remained unchanged after adjusting for education.

### Relationship between changes in functional connectivity and cognitive performance

In the stable group, we found an association between increased strength within the right supramarginal gyrus and improved performance on the BRB-N (*ß* = 0.18, 95% CI: 0.02, 0.32; *P* = 0.021). However, the reduction in FC graphs metrics in the declining group was more widely associated with BRB-N performance over time. In this group, reduced strength in the left accumbens and left paracentral regions (mean *ß* = 0.18, 95% CI: 0.04, 0.31; *P* = 0.012), as well as decreased local efficiency, primarily in the bilateral deep GM (mean *ß* = 0.17, 95% CI: 0.03, 0.3; *P* = 0.026) and right temporal cortex (mean *ß* = 0.16, 95% CI: 0.01, 0.29; *P* = 0.032), were linked to decreasing BRB-N *Z*-scores. Furthermore, a widespread reduction in the clustering coefficient, particularly in the bilateral deep GM (mean *ß* = 0.16, 95% CI: 0.03, 0.29; *P* = 0.023) and parietal cortex from both hemispheres (mean *ß* = 0.18, 95% CI: 0.04, 0.31; *P* = 0.015) was also associated with declining BRB-N performance (Fig. 2).

### Structural damage evolution

Whole brain volume and GM volume significantly decreased over time in both groups (GM volume in the stable group *P* = 0.001 and the declining group *P* < 0.001) at a similar rate (group differences for whole brain volume *P* = 0.438 and GM volume *P* = 0.761 at follow-up). Likewise, normalized lesion volume increased at follow-up in both groups (stable group *P* = 0.026 and declining group *P* = 0.003), also with a similar rate (*P* = 0.761). However, we found a specific increase in periventricular lesion volume in the cognitive worsening group (Supplementary Table 1).

**Figure 3:**
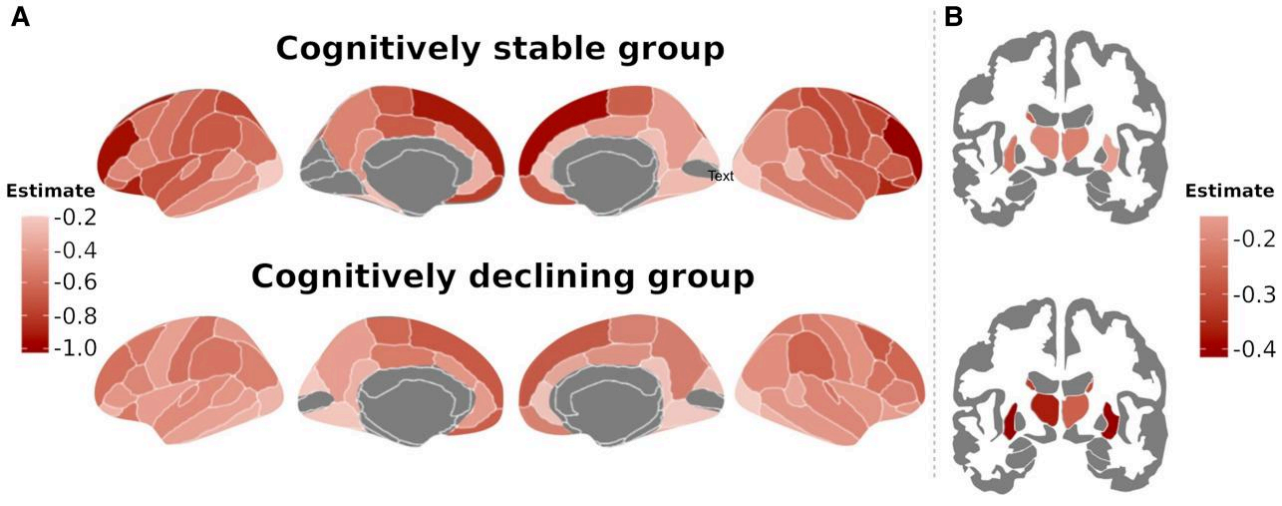
**Grey matter (GM) volumetric changes over study time.** The figure displays longitudinal differences in the volume of cortical and subcortical GM regions for the cognitively stable and cognitively declining groups analysed by mixed effect models with subject-specific random intercept (*P* < 0.05). The (**A**) panel shows cortical regions, and the (**B**) panel the subcortical GM regions. The intensity of the colour represents the magnitude of the volumetric changes, with darker red indicating larger differences. Figures were generated using *ggseg* (https://github.com/ggseg/ggseg) running in R Software.

At baseline, patients with declining cognitive performance showed a reduced regional GM volume in the left lateral orbitofrontal compared to the stable group (*Pcorr* = 0.012). During the study time, a decrease was observed in 60 (79%; mean *P* = 0.004) areas in cognitively stable patients, while the cognitively declined group showed a reduction in 64 (84.2%; mean *P* = 0.002) GM regions. However, the rate of regional volume changes over time differed between the two groups. Thus, left accumbens showed a higher rate of reduction in the cognitively declining group (*ß* = −0.40, 95% CI: −0.7, −0.1; *P* = 0.024), while frontal (bilateral orbitofrontal, left pars orbitalis and right superior frontal; mean *ß* = 0.42; 95% CI: 0.1, 0.7; *P* = 0.014) and left superior parietal cortex (*ß* = 0.41; 95% CI: 0.1, 0.7; *P* = 0.004) changed more in patients experimenting cognitive stability (Fig. 3).

## Discussion

This study demonstrates distinct trajectories of functional brain network topology in pwMS based on their cognitive performance. Using graph metrics, we quantified topographic properties of the functional network, stratifying pwMS into two groups according to their cognitive evolution. Results support the existence of two separate mechanisms of brain adaptation to damage, enhancing our understanding of the disease processes underlying cognitive impairment. Notably, patients with stable cognition showed an increase in such functional metrics over time, whereas patients who experienced global cognitive decline exhibited reduced connectivity, despite experiencing a similar increase in pathological structural brain burden. This suggests that patients with stable cognition were able to maintain FC, and consequently, preserve their cognitive performance.

We found that 64% of patients manifested global cognitive decline after a median of seven years. At baseline, both groups had comparable demographic, clinical and MRI characteristics, with mild disability (median EDSS of 2.0), predominantly relapsing-remitting form of the disease, and similar disease duration. At follow-up, both groups showed similar and significant reduction in GM volume and an increase in lesion volume. However, the response to this damage was different. While patients with stable cognitive function maintained or increased FC, those with cognitive decline exhibited widespread reductions in integration and segregation mechanisms, reflecting a loss of efficient communication within the functional network. Disruption of both brain connectivity and integration of central nodes has been linked to the progression of cognitive decline due to a decrease in network efficiency, suggesting a gradual depletion of beneficial adaptive mechanisms associated with disease progression.^32,33^

Longitudinal studies have highlighted both adaptive and maladaptive FC changes in relation to cognitive performance. Some studies reported increased coupling between structural and functional networks in patients with clinically isolated syndrome^34^ or increased connectivity within the ventral attentional network in patients with cognitive preservation or mild cognitive impairment who later experienced cognitive decline.^32^ Others observed a time-related reduction in FC within the default mode network in patients experiencing physical and cognitive deterioration over 2.5 years.^35^ Despite methodological differences, our findings and others support the conclusion that increasing MS disease burden leads to less flexibility in functional pathways, resulting in more rigid connectivity patterns and a progressive decline in FC,^32^ particularly in patients with worsening cognitive function.

Our findings also highlighted the involvement of specific brain regions associated with cognitive performance. Thus, increased strength in the right supramarginal gyrus was associated with better cognitive outcome, suggesting a compensatory role. More importantly, impaired integration and segregation of FC in core regions, such as deep GM, parietal and temporal lobes, were related with cognitive decline. These results reinforce existing theories that the ability to maintain both functional integrity and regional specialization over time might be among the main factors responsible for the preservation of cognitive functions.^9^ In the healthy brain, the functional brain network is hierarchically organized into interconnected subnetworks involved in cognitive processes. In MS, these subnetworks become more structurally segregated due to the vulnerability of long-range connections essential for integrating information across regions.^36^ This segregation worsens with disease progression, and correlates with cognitive deficits. Indeed, as structural brain damage accrual, adaptive mechanisms are required to compensate for this burden, which appears to differ according to cognitive performance. While both groups showed reduced GM and increased lesion volumes, only the cognitively stable group appeared to adapt functionally. In contrast, those with cognitive decline exhibited maladaptive changes, with a more random and disorganized network topology. Neuroplasticity and functional reorganization in response to brain damage are intrinsic properties of the CNS, such mechanisms include molecular changes, syntaptogenesis, synapsis and dendrite and axon sprouting changes^37^ that for unknown reasons may be more present in people able to maintain cognitive performance despite the accumulation of structural damage.^38^ Differences in disease expression may also be driven by patient-specific factors such as gender, age, social and environmental exposures, and genetic factors.^37^ The two groups showed similar demographic and clinical characteristics, including education as a common but limited proxy of cognitive reserve, highlighting the impact of other aspects on cognitive manifestations.

This study has strengths and limitations. One of the main strengths is the long-term follow-up in a well-characterized cohort of pwMS with comparable baseline clinical and demographic features, allowing for a robust analysis of FC changes over time relative to cognitive performance. The use of harmonized MRI protocols and graph metrics analysis further strengthens our findings. The study also has some limitations. The study is limited by the relatively small sample size and the absence of a comprehensive cognitive reserve assessment, relying only on education, prevented us from exploring its influence on the trajectories of FC networks. The absence of a healthy control group limits the ability to contextualize the results in relation to age-related cognitive and brain changes. Future studies including healthy participants with similar demographics are needed to reinforce the interpretability of disease-specific changes from age-related modifications. The inclusion of additional cognitive and MRI assessments would provide further insights into different cognitive trajectories and underlaying functional reorganization during the course of the disease. Finally, studies should combine diffusion-based MRI, as a measure of structural integrity, and analyse its influence on FC to shed light on the compensatory mechanisms that arise in response to accumulating brain damage.

In conclusion, pwMS show distinct trajectories of FC linked to their ability to cope with structural damage, impacting mid-term cognitive outcomes. Over seven years, those with cognitive decline exhibited reduced hierarchical segregation and integration, suggesting a maladaptive response. Whereas, cognitively stable patients maintained or increased connectivity despite similar structural brain burden. The underlying reason for these differing responses remains unclear and warrants further investigation.

## Supplementary Material

fcaf489_Supplementary_Data

## Data Availability

Anonymized data was not published in the article and can be shared on reasonable request from an investigator.
