# Supplementary material for: Cognitive outcomes in multiple sclerosis are shaped by divergent functional connectivity trajectories
Source: Brain Commun. 2025 Dec 12;8(1):fcaf489. doi: 10.1093/braincomms/fcaf489 (PMC12781869; doi:10.1093/braincomms/fcaf489)
Supplement: fcaf489_Supplementary_Data [file fcaf489_supplementary_data.docx]

**Supplementary material**

The acquisition included two 3D-Magnetization Prepared Rapid Acquisition Gradient Echo (3D-MPRAGE) structural sequences: (1) TR = 1800 ms; TE = 3.01ms; TI = 900ms; 240 sagittal slices; 0.94 mm isotropic voxel size; and a matrix size of 240 × 240 mm and (2) TR = 1970 ms; TE = 2.51 ms; TI = 1050 ms; 208 sagittal slices; 0.86 mm isotropic voxel size; and a matrix size of 220 × 220 mm. Additionally, two 3D-Fluid Attenuated Inversion Recovery (3D-FLAIR) protocol settings were used: (1) TR = 5000 ms; TE = 397 ms; TI = 1800 ms; 192 sagittal slices; 0.94 mm isotropic voxel size; and a matrix size of 240 × 240 mm and (2) TR = 5000 ms; TE = 303 ms; TI = 1800 ms; 208 sagittal slices; 0.86 mm isotropic voxel size; and a matrix size of 220 × 220 mm. The resting-state functional MRI images were acquired using the same acquisition protocol. We employed a BOLD EPI pulse sequence with fat saturation, with the following parameters: TR = 2000 ms; TE = 19 ms; field of view = 220 mm; 40 contiguous axial slices with 1.7 × 1.7 × 3 mm voxel size; GRAPPA-factor of 4; and a total of 450 frames. During this scan, participants were instructed to lie awake with their eyes closed and to think nothing in particular.

**Supplementary Table 1 Demographic, clinical and cognitive differences between baseline and follow-up by patient group according to cognitive performance.**

|  | **Stable group** | | | **Declining group** | | |
| --- | --- | --- | --- | --- | --- | --- |
|  | **baseline** | **follow-up** | ***P*-value** | **baseline** | **follow-up** | ***P*-value** |
| MS phenotype (n, %):  Relapsing-remitting  Progressive | 19 (90)  2 (10) | 18 (86)  3 (14) | 0.088 | 34 (94)  3 (6) | 31 (84)  6 (16) | **0.013*** |
| Median EDSS (range) | 2.0 (1.0, 6.5) | 2.0 (0, 8.0) | 0.459 | 2.0 (1.0, 6.0) | 2.0 (0, 7.5) | 0.248 |
| Use of DMT, (n, %)  None  Platform therapies  High efficacy | 6 (29)  14 (66)  1 (5) | 4 (19)  13 (62)  4 (19) | n.s. | 9 (24)  24 (65)  4 (11) | 12 (32)  22 (60)  3 (8) | n.s. |
| Percent of brain volume change (cm3) | – | -2 (-2.9, -1.5) |  | – | -2.9 (-3.4, -2) | – |
| GM volume (cm3) | 731.2  (683.8, 755.7) | 709.9  (693.2, 755.7) | **0.001*** | 729.4  (709.1, 755.1) | 705.2  (677.6, 739.3) | **<0.001*** |
| Lesion volume (cm3) | 3.3 (2.6, 16.3) | 5.2 (3.4, 17.7) | **0.026*** | 4.1 (2.8, 10) | 7.8 (4.2, 13.2) | **0.003*** |
| Periventricular (cm^3^) | 2.1 (1.4, 7.6) | 3.2 (1.2, 12.9) | 0.054 | 2.4 (1.1, 6.3) | 5.5 (2.2, 11.4) | **<0.001*** |
| Juxtacortical (cm^3^) | 0.4 (0.2, 1.4) | 0.6 (0.3, 1.4) | 0.871 | 0.6 (0.2, 1.7) | 0.6 (0.3, 1.9) | 0.096 |
| Subcortical (cm3) | 0.3 (0.2, 0.5) | 0.3 (0.2, 0.7) | 0.556 | 0.4 (0.2, 0.8) | 0.3 (0.2, 0.7) | 0.507 |
| BRB-N, z-score | -0.399  (-0.87, 0.4) | 0.155 (-0.48, 0.71) | **0.001*** | 0.092  (-0.28, 0.57) | -0.294  (-0.78, 0.57) | **<0.001*** |
| Verbal z-core | 0.556  (-0.62, 1) | -0.157  (-1, 0.53) | 0.350 | 0.370  (-0.14, 1.03) | -0.833  (-1.67, -0.33) | **<0.001*** |
| Visuospatial z-score | -0.625  (-1.5, 0.5) | 0.7  (-0.6, 1) | **0.003*** | 0.167  (-0.75, 0.75) | -0.25  (-0.92, 0.13) | **0.031*** |
| IPS-attention z-score | 0.321  (-0.3, 0.63) | 0.556  (0.28, 1.18) | **0.005*** | 0.359  (-0.021, 0.7) | 0.25  (-0.38, 0.79) | 0.148 |
| Verbal fluency z-score | -0.167  (-1, 0.14) | 0  (-1.14, 0.17) | 0.211 | -0.333  (-1, 0.14) | -0.667  (-1.3, 0) | **0.008*** |

Continuous variables are given as median and interquartile range, and qualitative data as numbers and proportion. BRB-N, Brief Repeatable Battery of Neuropsychological test; EDSS, Expanded disability status scale; IPS, information processing speed; GM, normalised grey matter.

Differences analysed by mixed effect models, except for the categorical variables, that we used multinomial logistic regression model. Variables are given as median and interquartile range, and qualitative data as numbers and proportion. Bolded values indicate statistically significant results (*P* < 0.05); n.s., not significant.
